# Impact of vitamin D in children with chronic tonsillitis (immunohistochemical study of CD68 polarisation and proinflammatory cytokines estimation)

**DOI:** 10.1038/s41598-023-33970-x

**Published:** 2023-05-17

**Authors:** Ayat Abu-elnasr Awwad, Rehab A. Hasan, Mohamed Ghazy Attia Hablas, Osama Mohammad Mohammad Abdelhay, Yahia Mohmmed Ahmed Dawood, Bothina Ahmed Mohamed, Khadiga Abdallah Abd Rabou, Taghreed Mahmoud Mohamed Salem, Marwa Elhady, Gehad Nabil Abd El-Aal, Ahmed Helal Elsayed Ahmed, Ahmed Ibrahim Mostafa Hasan, Asmaa Abd Elsalam Elmadbouly, Mohamed Basiouny Yahia, Walaa Mohamed Omar Ashry, Said S. M. M. El Sayed, Ashraf M. M. Algendy, Ahmad M. F. Alkot, Mohamed F. Farag, Ashraf Abdel Aty El Shenawy Emara, Fayez Mohammed Abd Elfattah Elbayoumy, Hany Fawzy Ali, Mohamed Morshdy Aldesoky, Raafat Abd-Rabow Abd-Eltwab, Samia M. Manawy, Eman Mohamed Faruk

**Affiliations:** 1 Department of otorhinolaryngology, Faculty of Medicine for Girls (AFMG), Al-Azhar University, Cairo, Egypt; 2 Department of Histology and Cell Biology, Faculty of Medicine for Girls (AFMG), Al-Azhar University, Cairo, Egypt; 3 Department of Histology and Cell Biology, Faculty of Medicine, Suez University, Suez, Egypt; 4 Department of Medical Physiology, Faculty of medicine, Al-Azhar University, Cairo, Egypt; 5 Department of otorhinolaryngology, Faculty of Medicine, Al-Azhar University, Cairo, Egypt; 6 Department of Pediatrics, Faculty of Medicine for Girls (AFMG), Al-Azhar University, Cairo, Egypt; 7 Department of Pediatrics, Faculty of Medicine, Al-Azhar University, Cairo, Egypt; 8 Department of Clinical Pathology, Faculty of Medicine for Girls (Cairo), Al-Azhar University, Cairo, Egypt; 9 Department of clinical pathology, Faculty of Medicine for Boys (Cairo), Al-Azhar University, Cairo, Egypt; 10 Department of Medical Microbiology and Immunology, Damietta Faculty of Medicine (girls), Al-Azhar University, Damietta, Egypt; 11 Department of Physiology, Armed Forces College of Medicine, Cairo, Egypt; 12 Department of Anatomy and Embryology, Faculty of Medicine, Al-Azhar University, Cairo, Egypt; 13 Department of Medical Microbiology and Immunology, Damietta Faculty of Medicine, Al-Azhar University, Damietta, Egypt; 14 Department of Anatomy and Embryology, Faculty of Medicine, Benha University, Benha, Egypt; 15 Department of Anatomy, Faculty of Medicine, Umm Al-Qura University, Makkah, Saudi Arabia

**Keywords:** Cell biology, Chemical biology

## Abstract

Inflammatory processes are increasingly attributed to macrophage polarization. Proinflammatory macrophages promote T helper (Th) 1 response, tissue repair, and Th2 responses. Detection of macrophages in tissue sections is facilitated by CD68. Our study is focused on the expression of CD68 and the estimation of proinflammatory cytokines in children’s patients with chronic tonsillitis secondary to vitamin D supplementation. This hospital-based Randomized prospective case–control study was conducted on 80 children with chronic tonsillitis associated with vitamin D deficiency where (40 received vitamin D 50,000 IU weekly for 3–6 months and 40 received 5 ml distilled water as placebo). The serum 25-hydroxyvitamin D [25(OH)D] was measured using an Enzyme-linked immunosorbent assay on all included children. Different histological and immunohistochemical studies for the detection of CD68 were done. There was a significantly lower serum level of 25(OH)D in the placebo group versus the vitamin D group (*P* < 0.001). The levels of pro-inflammatory cytokines, TNFα, and IL-2 significantly increased in the placebo group as compared to the vitamin D group (*P* < 0.001). The increased level of IL-4 and IL-10 in the placebo group as compared to the vitamin D group was insignificant (*P* = 0.32, 0.82) respectively. Vitamin D supplementation alleviated the deleterious effect of chronic tonsillitis on the histological structure of the tonsil. Tonsillar tissues of the children in the control and vitamin D groups demonstrated a highly statistically significantly lower number of CD68 immunoexpressing cells compared with those in the placebo group (*P* < 0.001). Low vitamin D may play a role in chronic tonsillitis. Vitamin D supplementation could help reduce the occurrence of chronic tonsillitis in susceptible children.

## Introduction

The human palatine tonsils are secondary lymphatic organs formed from a collection of lymphatic tissue. They have a significant role in the immune system of the body, in addition, they protect the mucosa of the alimentary tract against various pathogens. The paired palatine tonsils constitute the main lymphoid components in the lymphatic Waldeyer ring. This ring comprises pharyngeal tonsils (adenoids), lingual tonsils, and palatine tonsils. Waldeyer’s lymphoid tissue ring is also functionally called “mucosa-associated lymphoid tissue” or MALT and is the first line of adaptive defense against inhaled or ingested antigens^1^.

The most common age group for tonsillitis is children over 2 years old. Kids between the ages of 5 and 15 are more likely to develop tonsillitis caused by bacteria. Younger children are more prone to tonsillitis caused by viruses^2^. Several environmental factors contribute to recurrent pharyngotonsillitis, including poor sanitation, a high rooming index, a lack of sun exposure, and poor health habits^3^.

The tonsillar disease is a common cause of morbidity in children^2^. The choice of treatment is often tonsillectomy, which is still the most frequently performed surgical procedure in children^3^. Surgery can be associated with significant morbidity and very rare mortality, the only other reasonable therapeutic options for recurrent or chronic tonsillitis are repeated courses of antibiotics^2^.

Vitamin D, a fat-soluble vitamin, is synthesized in the skin upon sunlight and obtained from foods. Low vitamin D levels have been linked to many risk factors, including obesity, limited exposure to sunlight, prematurity, malabsorption, darkly pigmented skin, aging, chronic use of steroids or anticonvulsants, and low socioeconomic status^4^. In addition, several studies have reported that vitamin D deficiency may increase the risk of numerous acute/chronic otorhinolaryngologic conditions^2,5^.

The epithelial surfaces of the tonsils are modified to provide a large contact area for the capturing and presentation of antigens to the associated B- and T lymphoid stem cell populations^1^.

Fibrogenic cytokines secreted by activated macrophages or T lymphocytes are very important in the development of fibrotic disorders^6^. Circulating monocytes are attracted to tissues by chemotactic factors and become macrophages under the influence of their microenvironment^7,8^.

CD68 is a 110 kDa transmembrane glycoprotein expressed by human monocytes and macrophages. CD68 can be used for identifying a population of cells of mononuclear phagocyte origin, and for assessing the number of macrophages infiltrating a neoplasm. CR3/43 is the MHC Class II specific monoclonal antibody (mAb). CR3/43 is directed against the β -a chain of all products of the MHC class II gene subregions HLA-DR, HLA-DQ, and HLA-DP for microglial cells after antigen presentation^9^.

We aimed in this study to assess the potential effect of serum vitamin D levels in children’s patients with chronic tonsillitis based on the histological, immunohistochemical study of CD68, and the estimation of proinflammatory cytokines.

## Patients and methods

### Study design

This prospective case–control study was conducted from March 2020 to March 2022 at the otolaryngology department of Al-Zahraa Hospital, Al-Azhar University, Cairo, Egypt. The study was conducted under the Declaration of Helsinki and approved by the Local Ethics Committee of Al-Azhar University, Faculty of Medicine for Girls after pre-operative written informed consent from all children’s parents (IRB number 1625).

### Study population

This study includes three randomized groups; eighty children with chronic tonsillitis associated with vitamin D deficiency (vitamin D level < 20 ng/ml) were enrolled in the study and divided randomly into two groups according to the administration of vitamin D supplements.1- Vitamin D group: includes 40 children, aged 4 to 8 years old (23 boys,17 girls) who received oral Vitamin D 50,000 IU weekly, in the form of cholecalciferol by Gradual replenishment regimen continued for 3–6 months. (The course is completed until confirmed normal laboratory measurement of serum 25-hydroxyvitamin D [25(OH)D] level). 2-Placebo group: includes 40 children (23 boys,17 girls), aged 4 to 8 years old who received a placebo drug nearly of same course and duration as in the vitamin D group (5 ml distilled water). 3- Control group: includes 40 normal tonsillar biopsies taken from children with the normal level of 25(OH)D during adenoidectomy operations (these normal tonsils were based on clinical history and examination by laryngoscope before operation). They were aged 4 to 7 years old and were 23 boys and 17 girls.

### Diagnostic criteria of chronic tonsilitis

Tonsillitis is considered frequent when there have been seven episodes in the past year. Over the past 2 years, there have been at least five episodes a year. There have been at least three episodes per year in the past three years.

### Exclusion criteria

Children with renal impairment, mucociliary dysfunction, immune deficiency, and those with any contraindication for surgery. Also, any participants were taking any medications during the study.

### Laboratory investigations

Routine investigations were done for all included children in the form of complete blood count (CBC) (Sysmex KX21N, Kobe, Japan). ESR (Westergren method) Coagulation profile (Stago, France), liver function, and kidney function (Cobas c 311, Roche, Germany).

Measurement of serum 25(OH)D level using the enzyme-linked immunosorbent assay (ELISA) kit (Calbiotech, USA, California Cat# VD220B) according to the manufacturer's instructions. The detection range of the kit is 2.5–150 ng/ml. Each sample was run in duplicate and compared with a standard curve. The mean concentration was determined for each sample. Vitamin D deficiency was defined as 25(OH)D below 20 ng/ml, vitamin D insufficiency as 25(OH)D of 21–29 ng/ml, and normal 25(OH)D level as ≥ 30 ng/ml^10^.

Blood samples were collected and centrifuged to separate serum from all groups. IL-2 (Elabscience, cat. No. E-El-H0099), TNF-α (Elabscience, cat. No. E-EL-H0109), IL-10 (Elabscience, Cat. No. E-El-H0103), and IL-4 (Elabscience, cat. No. E-El-H0101) were estimated using enzyme-linked immunosorbent assay (ELISA) technique and according to the manufacturer.

Tonsillectomies were performed in the otolaryngology department of Al-Zahraa Hospital, Al-Azhar University, Cairo, Egypt, and outpatient clinics.

### Histological study

All the tonsillar specimens were fixed in 10% formol saline for 24 h, washed, then exposed to serial concentrations of ethyl alcohol, and ended with absolute alcohol (for complete dehydration). Specimens were cleared in xylene and embedded in paraffin for 24 h. The rotary microtome prepared paraffin wax tissue blocks for sectioning at 4 μm thicknesses (LEICA RM 2125 UK). The obtained tissue sections were collected on glass slides, deparaffinized, and stained with H&E^11^ and Sirius red^12^. H&E stain to assess the general histological structure of palatine tonsil while Sirius red is used to assess collagen fibers deposition (fibrosis).

Slides were examined under a light microscope (Primo star, ZEISS, China). The photos were taken using (an Axiocam ERc 5 s, ZEISS, China) camera, at the Histology Department, Faculty of Medicine for Girls, Al Azhar University.

### Immunohistochemical study

Palatine tonsil sections were processed and immunostained using peroxidase-labeled streptavidin–biotin for CD68 (Monoclonal Mouse Anti-Human CD68, clone PG-M1 DAKO-Denmark, diluted 1:50) for macrophages^13^. Positive slide was provided by the manufacturer. Negative control sections were prepared with the omission of the primary antibody.

### Morphometric study

- Area% of the stained collagen fibers around lymphatic follicles and in the subepithelial region in 10 randomly selected fields per group at × 40 magnification in Sirius red-stained sections. The results were expressed as mean area % of collagen/µm^2^ ^12^. This parameter was measured using a computerized image system composed of a Leica Qwin 500 image analyzer connected to a Leica microscope in Cambridge (UK).
- CD68 immunoexpressing appeared as a brown cytoplasmic reaction. The number of CD68 positive cells/ high power field was measured in ten fields per group using the same system and expressed as cell number per µm^2^ ^14^.

### Statistical analysis

Continuous variables were expressed as mean ± standard deviation (SD) or median and range. While categorical variables were expressed as numbers and percentages. Shapiro–Wilk test was used to assessing the normality of the continuous variables while Bartlett’s test was used to assess the equality of variance. The ordinary one-way analysis of variance (ANOVA) test was performed to detect statistical differences among the study groups. posthoc Tukey's multiple comparisons test was performed for multiple comparisons between the study groups. Differences were considered significant at a *P* < 0.05. All statistical comparisons were two-tailed. All statistical analyses were calculated by GraphPad Prism, Version 8.0 Software (GraphPad Software; SanDiego, CA, USA).

### Ethics approval

The study was conducted under the guidelines for the ethical treatment and handling of patients in research. All the experiments were approved by the Local Ethics Committee of Al-Azhar University, Faculty of Medicine for Girls after pre-operative written informed consent from all children's parents. Consent to participate is Applicable.

### Consent to participate 

Applicable.

## Results

### Clinical results

A total of 80 children with chronic tonsillitis were recruited in the study, all aged 4 to 8 years with a mean (SD) age of 5.4 (1.1) years, 58% were boys. (Table 1).

**Table 1:** Baseline characteristics of the participants.

Characteristics	Total *n* = 120	Control *n* = 40	Vitamin D *n* = 40	Placebo *n* = 40	*P*-value‡
Age (years)	5.4 ± 1.1 5(4–8)	5.6 ± 1.0 5(4–7)	5.2 ± 1.0 5(4–7)	5.5 ± 1.2 5(4–8)	0.13
Boys *n* (%)	69(58)	23(58)	23(58)	23(58)	0.18
Girls *n* (%)	34(43)	17(43)	17(43)	17(43)	0.27
*P*-value†			< 0.001	0.951	

Data are mean ± SD or median(minim-maximum) unless otherwise stated. The percentage is not total to 100 because of rounding.

The vitamin D group showed a highly statistically significant elevation in serum vitamin D concentration 3–6 months following vitamin D supplementation compared with its initial concentration (*P* < 0.001) (Table 2).

**Table 2:** Serum vitamin D concentration at baseline and 3 months following administration of the participants.

Groups	Serum vitamin D concentration (ng/ml)		*P*-value†
	At baseline	After 3–6 months	
Vitamin D group	12.5 ± 5.3	37.9 ± 6.3	< 0.001
Placebo group	12.6 ± 4.6	12.7 ± 5.6	0.95
*P*-value‡	0.95	< 0.001	

Data are mean ± SD.

^†^Paired samples t-test (same group).

^‡^Independent samples-test (between two groups).

The levels of pro-inflammatory cytokines, TNFα, and IL-2 appear to be increased significantly in patient groups with placebo as compared to the vitamin D group (Table 3). An increase in the level of IL-4 and IL-10 was also observed in the placebo group as compared to the vitamin D group, yet this increase was not significant (*P* = 0.32, 0.82) respectively.

**Table 3:** levels of TNFα, IL2, IL4 and IL10 (pg/ ml) in different studied groups.

Groups	Control	Vitamin D	Placebo	*P*-value‡
TNFα	22.1 ± 0.4	20.3 ± 0.6	54.5 ± 8.2	< 0.001
IL-2	16.3 ± 3.0	18.1 ± 9.1	89.5 ± 11.5	< 0.001
IL-4	20.1 ± 6.3	23.1 ± 8.7	29.1 ± 44.7	0.32
IL-10	10.5 ± 2.2	12.3 ± 1.9	19.29 ± 2.0	0.4
*P*-value†		< 0.001	0.82	

Data are mean ± SD.

^†^Paired samples t-test.

^‡^Independent sample-test.

### Histological results

#### H&E

**Figure 1:**
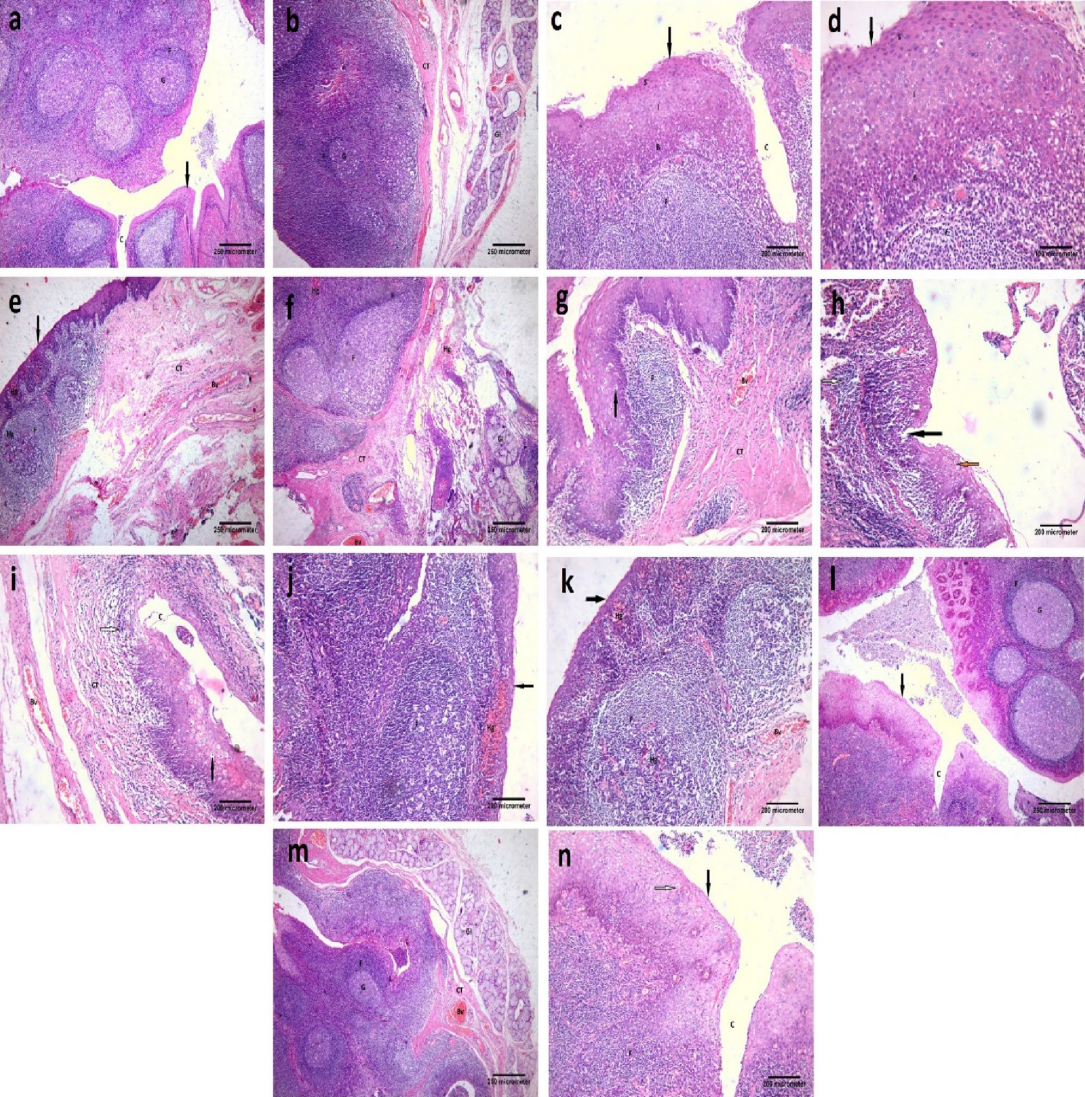
Photomicrographs of palatine tonsil sections stained with H&E showing: (**a**) Control group, covered with stratified squamous nonkeratinized or para keratinized surface epithelium (arrow). This epithelium is thrown into invaginations called crypts (C). There are many lymphatic follicles (F) in the subepithelial region. These follicles have a peripheral darkly stained zone (mantle zone or corona) and central pale stained zone; germinal center (G) [× 40, scale bar = 250 µm]. (**b**) Control group, there is a fibrous CT band (CT) surrounding the base of the palatine tonsil. Under this CT, there are numerous glands (Gl). Within the tonsil, multiple lymphatic follicles (F) can be seen with germinal center (G) [× 40, scale bar = 250 µm]. (**c**) Control group, showing the covering epithelium (arrow) of palatine tonsil. This epithelium is formed of the basal layer (B), intermediate layers (I), and superficial layer (S). Notice, the crypt (C) and lymphatic follicle (F) [× 100, scale bar = 200 µm]. (**d**) A higher magnification of the previous section showing the basal layer of the epithelium (B). It is formed of columnar cells with basal oval nuclei. Intermediate layers (I), multiple layers of polyhedral cells with central rounded nuclei. Superficial layer (S), multiple layers of flat cells with flat nuclei. Notice, the lymphatic follicle (F) which is formed of aggregation of lymphocytes with darkly stained nuclei [× 200, scale bar = 100 µm]. (**e**) Placebo group, showing thinning of the covering epithelium (arrow) with multiple areas of hemorrhage (Hg) can be seen within it. An apparent decrease in the number of lymphatic follicles (F), some of them have areas of hemorrhage (Hg). An apparent increase in connective tissue (CT) under the epithelium with multiple dilated congested blood vessels (Bv) within it [× 40, scale bar = 250 µm]. (**f**) Placebo group, showing follicular expansion and irregularity (F). Thickening of the CT around the tonsil (CT). Multiple areas of hemorrhage (Hg) and dilated congested blood vessels (Bv) can be seen. Notice, the gland (Gl) [× 40, scale bar = 250 µm]. (**g**) Placebo group, showing multiple vacuolated epithelial cells (arrow) within the epithelium. An apparent decrease in the number of lymphatic follicles (F). The apparent increase in connective tissue (CT) under the epithelium with dilated congested blood vessels (Bv) within it [× 100, scale bar = 200 µm]. (**h**) Placebo group, showing an area of erosion; ulceration (black arrow) within the epithelium. Some vacuolated cells appear within the epithelium (orange arrow). A marked mononuclear cellular infiltration (white arrow) can be seen [× 100, scale bar = 200 µm]. (**i**) Placebo group, showing many vacuolated cells within the covering epithelium (black arrow). Crypt (C) can be seen with desquamated cells within it. Lymphatic follicles can't be seen. A mononuclear cellular infiltration (white arrow) can be seen within the CT tissue. Notice, multiple dilated congested blood vessels (Bv) [× 100, scale bar = 200 µm]. (**j**) Placebo group, showing thinning of the covering epithelium (arrow) with a large area of hemorrhage (Hg) can be seen within it. Enlargement of the lymphatic follicle (F) under the epithelium is observed [× 100, scale bar = 200 µm]. (**k**) Placebo group, showing thinning of the covering epithelium (arrow) with multiple areas of hemorrhage (Hg) can be seen within it. Enlargement of the lymphatic follicle (F) under the epithelium is observed. Areas of hemorrhage (Hg) can be seen within this follicle. Notice, multiple dilated congested blood vessels (Bv) [× 100, scale bar = 200 µm]. (**l**) Vitamin D group, showing that the palatine tonsil restores its histological architecture, it appears the same as the control group. Notice, the epithelial covering (arrow) with crypt (C) and lymphatic follicles (F) with germinal centers (G) [× 40, scale bar = 250 µm]. (**m**) Vitamin D group, showing that the palatine tonsil restores its histological architecture, it appears the same as the control group but there are some congested blood vessels (Bv). Notice, the lymphatic follicles (F) with germinal centers (G), CT band, and glands (Gl) [× 40, scale bar = 250 µm]. (**n**) Vitamin D group, showing that the palatine tonsil restores its histological architecture, appears the same as the control group. The epithelial covering (black arrow) has some vacuolated cells (white arrow). Notice, the crypt (C) and lymphatic follicle (F) [× 100, scale bar = 200 µm].

Examination of H&E-stained sections of the Control group revealed that the palatine tonsils are partially encapsulated lymphoid tissue consisting of dense accumulations of lymphatic tissue located in the mucous membrane. A well-organized stratified squamous nonkeratinized or para-keratinized epithelium (15–20 layers) covered the mucosal surface of the palatine tonsil. The squamous epithelium that forms the surface of the tonsil dips into the underlying CT in numerous places, forming tonsillar crypts. When the surface epithelium traced toward the crypt it started to lose its organization and become thinnest gradually. There are 10–20 crypts for tonsils. The walls of these crypts usually possess numerous lymphatic nodules. Under the epithelium, there is an interfollicular zone in which the lymphoid tissue is arranged into lymphoid nodules with germinal centers. Separating the lymphoid tissue from adjacent structures is a band of dense fibrous CT that acts as a capsule or barrier against spreading tonsil infections. Mucous glands are present in CT, their ducts open on the surface and not in the base of tonsillar crypts, so inflammation of the crypts is common. (Fig. 1a–d).

Examination of H&E-stained sections of the tonsils in the Placebo group before vitamin D supplementation indicated thinning of the covering epithelium with multiple areas of hemorrhage or surface epithelium erosions (ulceration). An apparent decrease in the number of lymphatic follicles, some of them had areas of hemorrhage. Sometimes, follicular expansion (enlargement) and irregularity could be seen. An apparent increase in the connective tissue under the epithelium with multiple dilated congested blood vessels within it could be observed. Marked mononuclear cellular infiltration could be seen within this CT tissue. (Fig. 1e–k).

In the patients treated with vitamin D, alleviation of all deleterious effects was observed. The examined sections revealed an apparent normal tonsillar structure. The palatine tonsils had restored their regular histological architecture. But there were some congested blood vessels, and the epithelial covering had some vacuolated cells. (Fig. 1l–n).

#### Sirius red stain

**Figure 2:**
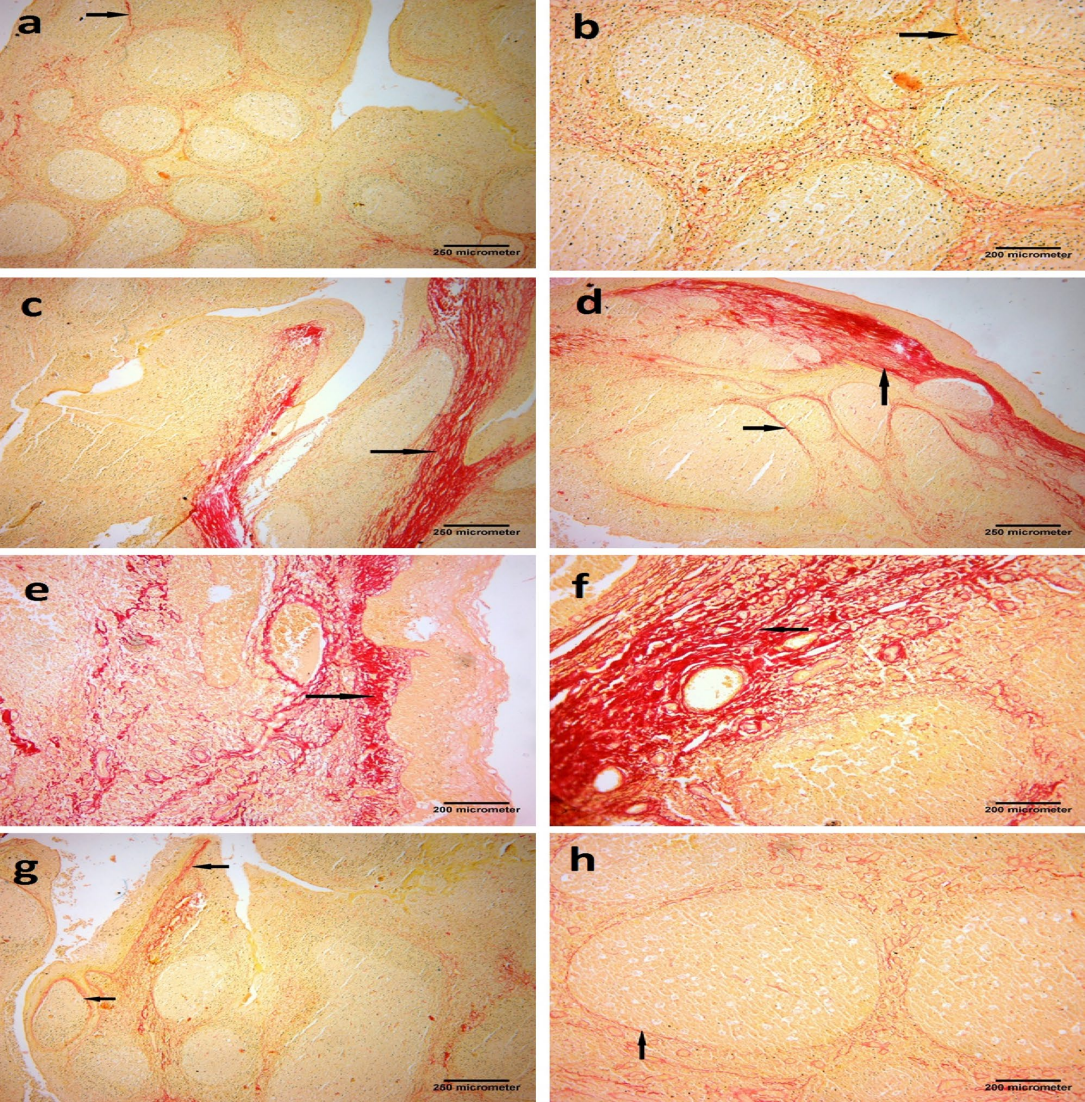
Photomicrographs of palatine tonsil sections stained with Sirius red stain showing: (**a**) Control group, minimal collagen fibers deposition around the follicles (arrow) [× 40, scale bar = 250 µm]. (**b**) Control group, few collagens’ fibers depositions around the follicles (arrow) [× 100, scale bar = 200 µm]. (**c**) Placebo group, thick collagen bands in the subepithelial region (arrow) [× 40, scale bar = 250 µm]. (**d**) Placebo group marked deposition of thick collagen bands in the subepithelial region and between the follicles (arrows) [× 40, scale bar = 250 µm]. (**e**) Placebo group, deposition of thick collagen fibers in the subepithelial region (arrow) [× 100, scale bar = 200 µm]. (**f**) Placebo group marked deposition of thick collagen fibers within the tonsil (arrow) [× 100, scale bar = 200 µm]. (**g**) Vitamin D group, mild collagen fibers deposition in the subepithelial region and around the follicles (arrows) [× 40, scale bar = 250 µm]. (**h**) Vitamin D group, mild collagen fibers deposition around the follicles (arrow) [× 100, scale bar = 200 µm].

In Sirius red–stained tonsillar sections, minimal collagen fibers (stained red) deposition was seen around the follicles in the Control group, while the tonsillar stroma appeared enriched of collagen fibers which organized in thick fascicles in the Placebo group before vitamin D supplementation. The vitamin D-treated group (group III) showed mild collagen fibers deposition in the subepithelial region and around the follicles. (Fig. 2).

#### Immunohistochemical results

**Figure 3:**
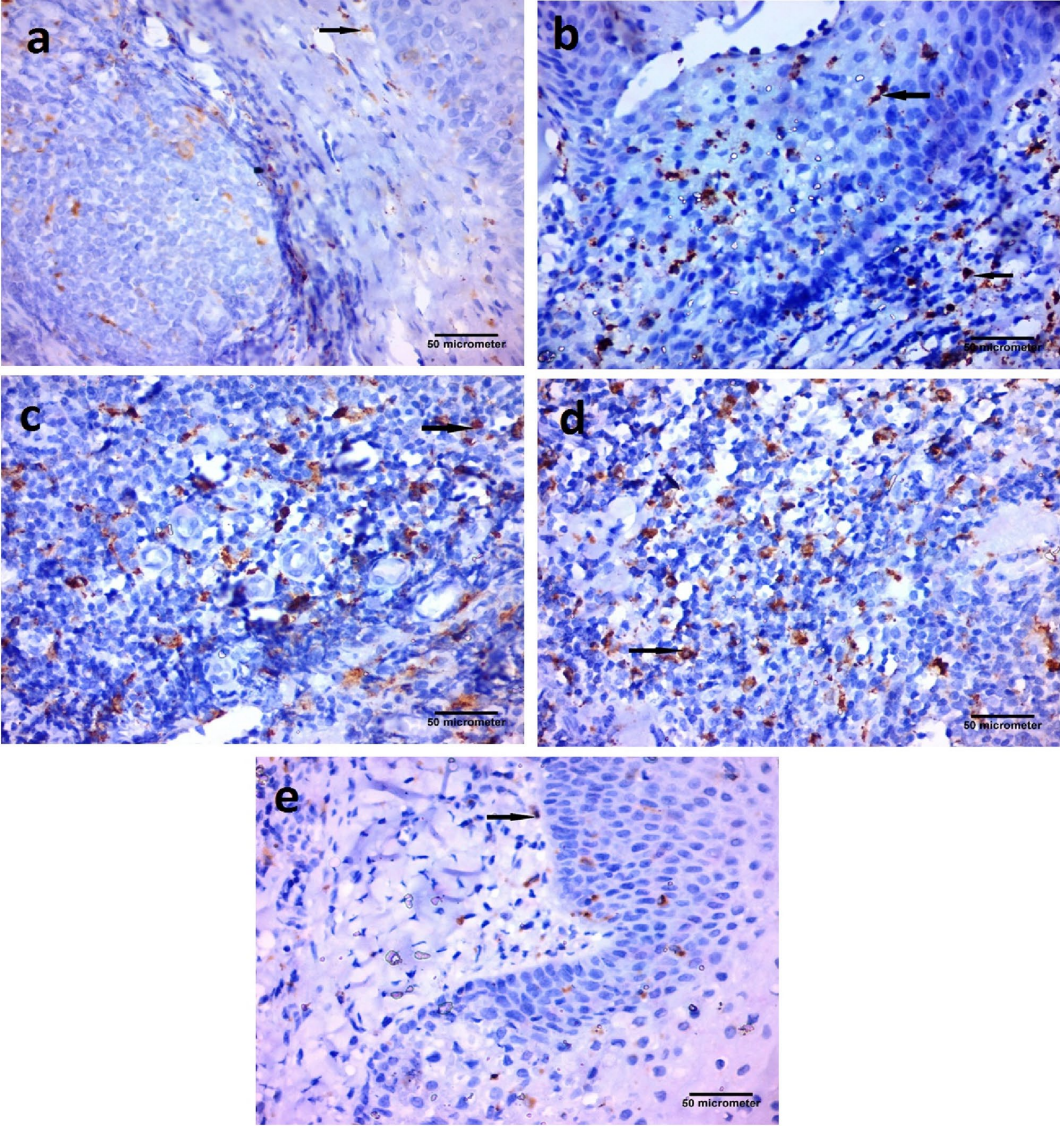
Photomicrographs of palatine tonsil sections stained with immunohistochemical stain for CD68 [Avidine biotin peroxidase stain with Hx counter stain X 400, scale bar = 50 µm] showing: (**a**) Control group, few CD68 positive cells, macrophages at the subepithelial level (arrow). (**b**) Placebo group, numerous CD68 positive cells, and macrophages present in the epithelial covering and at the subepithelial level (arrows). (**c**) Placebo group, numerous CD68 positive cells, macrophages at the subepithelial level (arrow). (**d**) Placebo group, numerous CD68 positive cells, macrophages at the subepithelial level (arrow). (**e**) Vitamin D group, some CD68 positive cells, macrophages at the subepithelial level (arrow).

CD68 immunoexpressing cells appeared as a brown cytoplasmic reaction. Immunostaining with CD68 revealed the presence of macrophages. Few CD68-positive cells and macrophages at the subepithelial level were detected in the Control group. In addition, some CD68-positive cells were demonstrated in the Vitamin D group. In contrast, numerous CD68-positive cells, and macrophages were detected in the epithelial covering and at the subepithelial level in the Placebo group. (Fig. 3).

### Morphometric results

**Figure 4:**
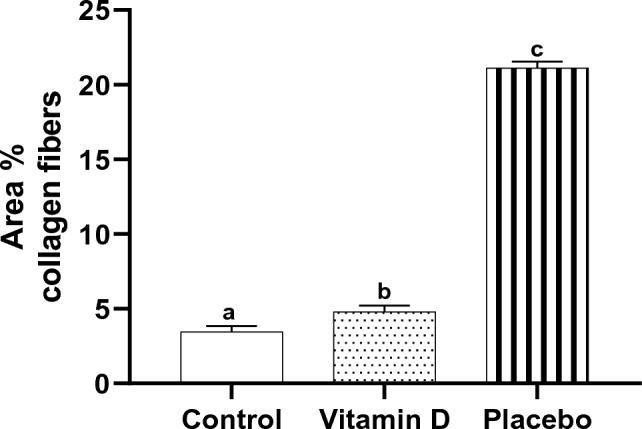
Effect of vitamin D supplementation on area percent collagen fibers in tonsillar tissue, *n* = 10. Bars not sharing the common superscript letters differ significantly at a *P* < 0.001 by ANOVA followed by post hoc Tukey's multiple comparisons tests.

Tonsillar tissues of the children in the control and vitamin D groups demonstrated a highly statistically significantly lower mean area percent of collagen fibers compared with those in the placebo group (3.5 ± 0.4, 4.8 ± 0.4, and 21.1 ± 0.5, respectively, *P* < 0.001). Whereas the mean area percent of collagen fibers of tonsillar tissues of the children supplemented with vitamin D children still showed a highly statistically significantly higher mean area percent of collagen fibers compared with those in the control group (*P* < 0.001) (Fig. 4).

**Figure 5:**
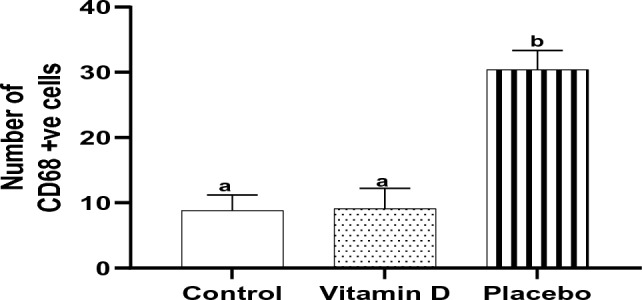
Effect of vitamin D supplementation on the number of CD68 + ve cells in tonsillar tissue, *n* = 10. Bars not sharing the common superscript letters differ significantly at a *P* < 0.001 by ANOVA followed by post hoc Tukey's multiple comparisons tests.

Tonsillar tissues of the children in the control and vitamin D groups demonstrated a highly statistically significantly lower number of CD68 + ve cells compared with those in the placebo group (8.8 ± 2.4, 9.1 ± 3.1 and 30.4 ± 3.0, respectively, *P* < 0.001 each). No statistically significant difference between the control and vitamin D groups was observed (*P* = 0.97) (Fig. 5).

## Discussion

The main findings of the present study revealed a significantly lower serum level of 25(OH)D in the Placebo group versus the Vitamin D group. Vitamin D deficiency was significantly more detected in children with chronic tonsillitis compared to children treated with vitamin D. This hypovitaminosis D increased the risk for chronic tonsillitis (*P* < 0.05).

As regards the histological examination of tonsils in the Placebo group before vitamin D supplementation, we found thinning of the covering epithelium with multiple areas of hemorrhage or surface epithelium erosions (ulceration). The apparent decrease in the number of lymphatic follicles, some of them had areas of hemorrhage. Sometimes, follicular expansion (enlargement) and irregularity could be seen. This follicular enlargement is consistent with Mogoanta et al.^14^ who stated that follicular enlargement is generated by the proliferation of B-lymphocytes which mostly occupied the germinal center.

Marked mononuclear cellular infiltration could be seen within tonsillar tissue. Also, there was a significant increase in collagen fibers deposition in the subepithelial region and around the lymphatic follicles.

Palatine tonsil, just like the entire Waldeyer lymphatic circle, contains an increased immunologic reactivity tissue, thus building a barrier against the pathogenic agents’ penetration into the respiratory and digestive tracts^15^. They take part both in humoral immunity by synthesis and secretion of a great number of immunoglobulins, which neutralize a part of the oropharynx cavity flora, and in cell immunity by T-lymphocytes penetrating the epithelial barrier^16^.

A common histological characteristic of chronic tonsillitis and tonsillar hypertrophy is immunological parameters, genetic susceptibility, and local lymphocyte dysfunction. The endoplasmic reticulum (ER) is an organelle that has a very important function in the balanced functioning of cells, in which the accumulation of a cellular protein called ER stress occurs in case of cellular dysfunction. ER stress influences the pathogenesis of many diseases and immune system functions. In a study by Onal, Merih, et al.^17^ it was found that endoplasmic reticulum ER stress response and apoptosis pathway are also important in the pathogenesis of chronic tonsillitis and tonsillar hyperplasia.

The palatine tonsils are covered with stratified squamous nonkeratinized epithelium. This epithelium is avascular and only a very few nonepithelial cells are found in a normal healthy person. In chronic infection as chronic tonsillitis, the circulation of blood is poor because of degenerative changes causing parenchymal fibrosis^5,18^. The fibrosis of the tonsillar tissue due to repeated tonsilitis result in damage to the barrier function of the tonsils with a local dysfunction of the immunity, which subsequently caused a persistent infection, at the same time inflammatory process succeed to proliferate and activates the fibroblastic producing collagen cells in addition to the immune cells to replace the immunologic active tissue with fibrous tissue. Consequently, regulating fibrogenic processes may be a significant therapeutic option in diseases associated with chronic inflammation^3^.

This study researches the association between vitamin D levels and inflammatory cytokines in children with chronic tonsillitis. Rendering to our results vitamin D had a significant association with pro-inflammatory cytokines (TNFα and IL-2) and a non-significant association with anti-inflammatory cytokines (IL-4 and IL-10). The correlation between vitamin D administration and serum levels of inflammatory markers such as cytokines has been investigated previously by Sun and colleagues^19^. Some studies have indicated that there is a direct correlation between IFNγ and IL-10^20^.

Immunostaining with CD68 revealed the presence of macrophages. In this study, subepithelial and intraepithelial disposed of macrophages were found in the Placebo group. Mogoanta et al., 2008^14^ stated that an intensely positive reaction to CD68 was also observed under the basal membrane of the covering epithelium and even in the structure of the epithelium. The macrophages' presence at that level is ordered by the existence of the pathogenic germs from the tonsil surface.

The results of the current study proved the potential effects of vitamin D in patients with chronic tonsillitis. The tonsillar sections restored their histological structure in the Vitamin D group. Vitamin D has an important role in maintaining immunity. Vitamin D has numerous effects on different cell types of the immune system such as dendritic cells, B lymphocytes, T lymphocytes, and NK cells^21^.

Vitamin D receptors are found in T cells, B cells, antigen-presenting cells, macrophages, and dendritic cells. Vitamin D immunomodulates both innate and adaptive immune responses^6^. In terms of the innate immune system, vitamin D increases the production of antimicrobial peptides (AMPs), including defensin ß and cathelicidin^22,23^. All of these provide a natural defense against potential pathogens. People suffering from recurrent tonsillitis have reduced amounts of the AMPs cathelicidin and defensin b2 on the tonsil surface and in the tonsillar crypt epithelium when compared with normal controls. Low AMP levels have been found in patients suffering from recurrent tonsillitis^4^. In the adaptive immune system, vitamin D inhibits the proliferation of activated lymphocytes, reduces the production of inflammatory cytokines, and promotes the development of induced regulatory T cells^6^. Several studies reported that vitamin D supplements had a protective effect against acute respiratory infection, particularly in patients with profound vitamin D deficiency^24,25^.

In terms of the effects of vitamin D on the adenoids and tonsils, a deficiency may increase recurrent infections. In addition, vitamin D regulates human tonsillar T cells, and a deficiency may trigger tonsillar hypertrophy^6^. A Turkish study by San et al.^5^ found that children with recurrent tonsillitis and allergic rhinitis had significantly lower 1,25-dihydroxy vitamin D [1,25(OH)2D] levels than controls.

Children with COVID-19 who have secondary lymphoid tissue such as tonsils may have a milder course of the disease^26^.

## Conclusion

In conclusion, our study has seen an association between vitamin D intake and the levels of TNF-α, some interleukins, and CD68 expression in children with chronic tonsillitis. Future studies should focus on larger populations, considering seasonal variations and standardizing tonsil size measurements. The prevalence of vitamin D deficiency is extremely high at present, which is why vitamin D screening should be encouraged for patients with recurrent tonsillitis.

## Data Availability

The data used and/or analyzed during this study are available from the corresponding author upon reasonable request.
